# Supplementary material for: Cryo-electron tomography reveals the microtubule-bound form of inactive LRRK2
Source: bioRxiv. 2024 Jun 20:2024.06.18.599606. Preprint. [Version 1] doi: 10.1101/2024.06.18.599606 (PMC11212993; doi:10.1101/2024.06.18.599606)
Supplement: Supplementary file 1 [file NIHPP2024.06.18.599606v1-supplement-1.pdf]

bioRxiv preprint doi: https://doi.org/10.1101/2024.06.18.599606; this version posted June 20, 2024. The copyright holder for this preprint (which was not certified by peer review) is the author/funder, who has granted bioRxiv a license to display the preprint in perpetuity. It is made available under aCC-BY 4.0 International license.

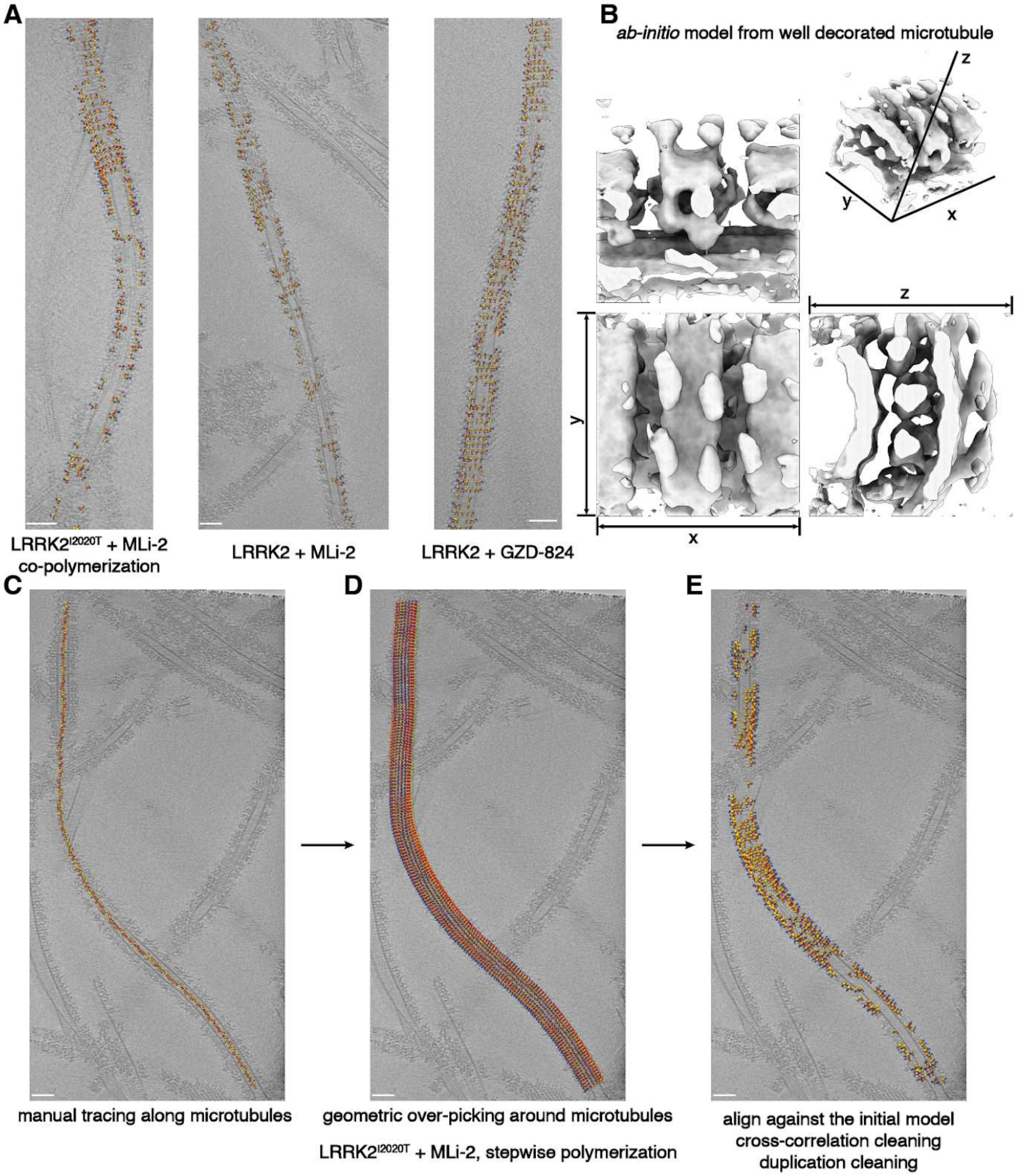

Figure S1: Geometry-based extraction of LRRK2-microtubule subtomograms. (A) Sample tomogram slices showing LRRK2-decorated microtubules from three cryo-ET datasets. The LRRK2 variant and kinase inhibitor used in each dataset are indicated. subtomograms that went into the final reconstruction are shown as well, overlayed on the tomogram slice in their final orientation and position. **(B)** Three orthogonal views of our initial LRRK2-microtubule map, with a reconstructed 3D map shown in the up-right corner. The corresponding x-y-z axis are labeled in the orthogonal views and the reconstructed map respectively. **(C-E)** Step-wised representation of LRRK2-microtubule subtomogram extraction workflow (see Methods). **(C)** Subtomograms are first regularly picked along microtubules to serve as reference points for LRRK2 subtomogram picking. Sample tomogram slice showing LRRK2-decorated microtubules from the LRRK2^{I2020T} + MLi-2 + microtubules dataset (with LRRK2^{I2020T} incubated with pre-assembled microtubules) is overlayed by microtubule subtomograms, in their final orientation and position. **(D)** The same tomogram slice overlapped with over-picked LRRK2 subtomograms regularly distributed around each microtubule subtomograms shown in (C). **(E)** The same tomogram slice overlapped with cleaned LRRK2 subtomogram subsets after duplication cleaning and cross-correlation based cleaning. Over-picked subtomograms and subtomograms that are poorly correlated with our initial model were cleaned out. Coordinates of subtomograms are labeled as orange spheres and their refined x-y-z orientations are shown as yellow-red-blue arrows. Scale bar: 50 nm.

bioRxiv preprint doi: https://doi.org/10.1101/2024.06.18.599606; this version posted June 20, 2024. The copyright holder for this preprint (which was not certified by peer review) is the author/funder, who has granted bioRxiv a license to display the preprint in perpetuity. It is made available under aCC-BY 4.0 International license.

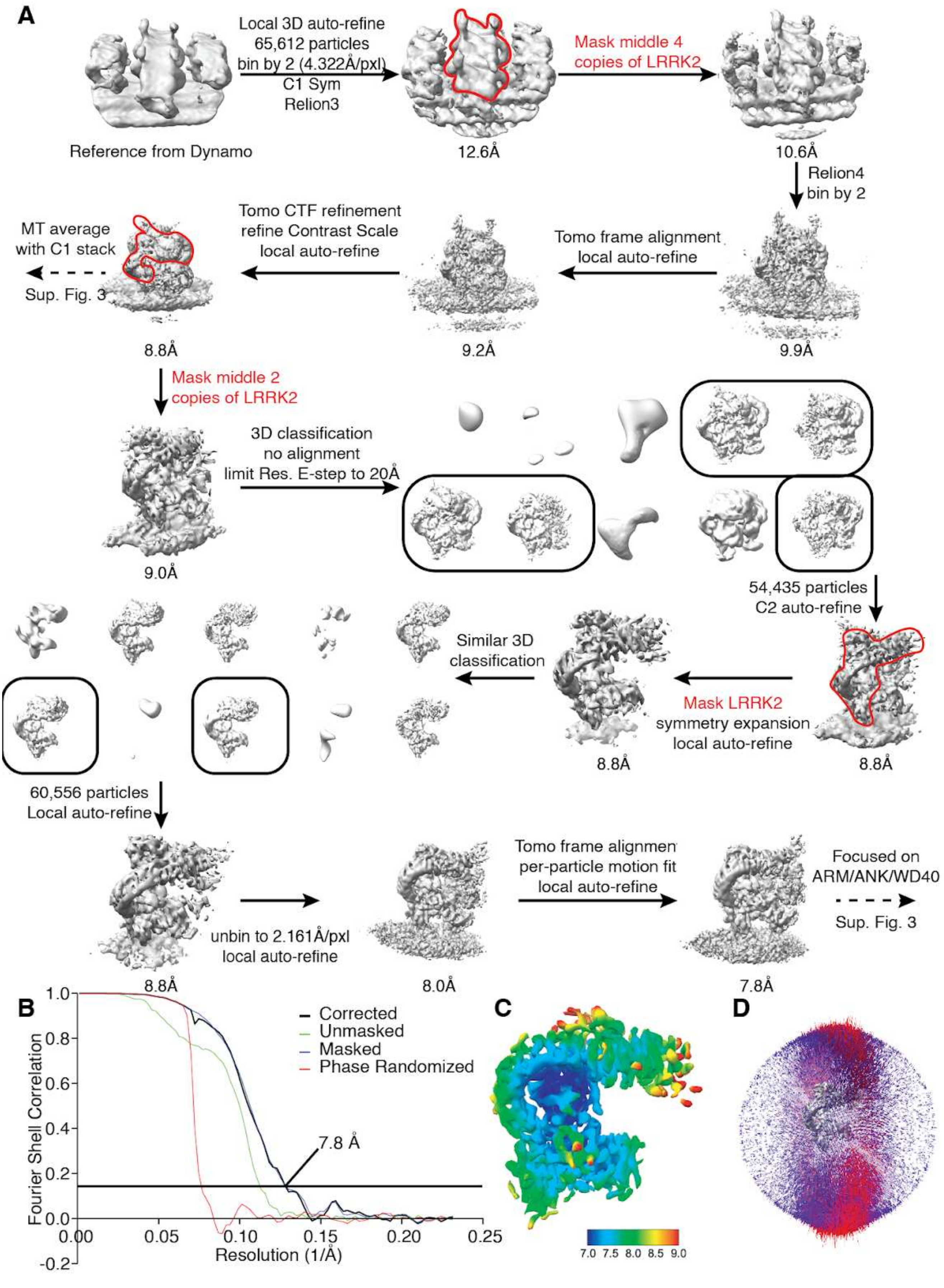

Figure S2: Data-processing scheme for the LRRK2^{I2020T} + MLi-2 + microtubule sample. (A) Flow chart of the cryo-ET data processing procedure. **(B)** The gold-standard Fourier Shell Correlation (FSC) curves (0.143 cutoff) show the final resolution of the LRRK2 focused-refinement map. **(C)** Local resolution of the LRRK2 focused-refinement map. **(D)** Particle projection angle distribution plot of the map.

bioRxiv preprint doi: https://doi.org/10.1101/2024.06.18.599606; this version posted June 20, 2024. The copyright holder for this preprint (which was not certified by peer review) is the author/funder, who has granted bioRxiv a license to display the preprint in perpetuity. It is made available under aCC-BY 4.0 International license.

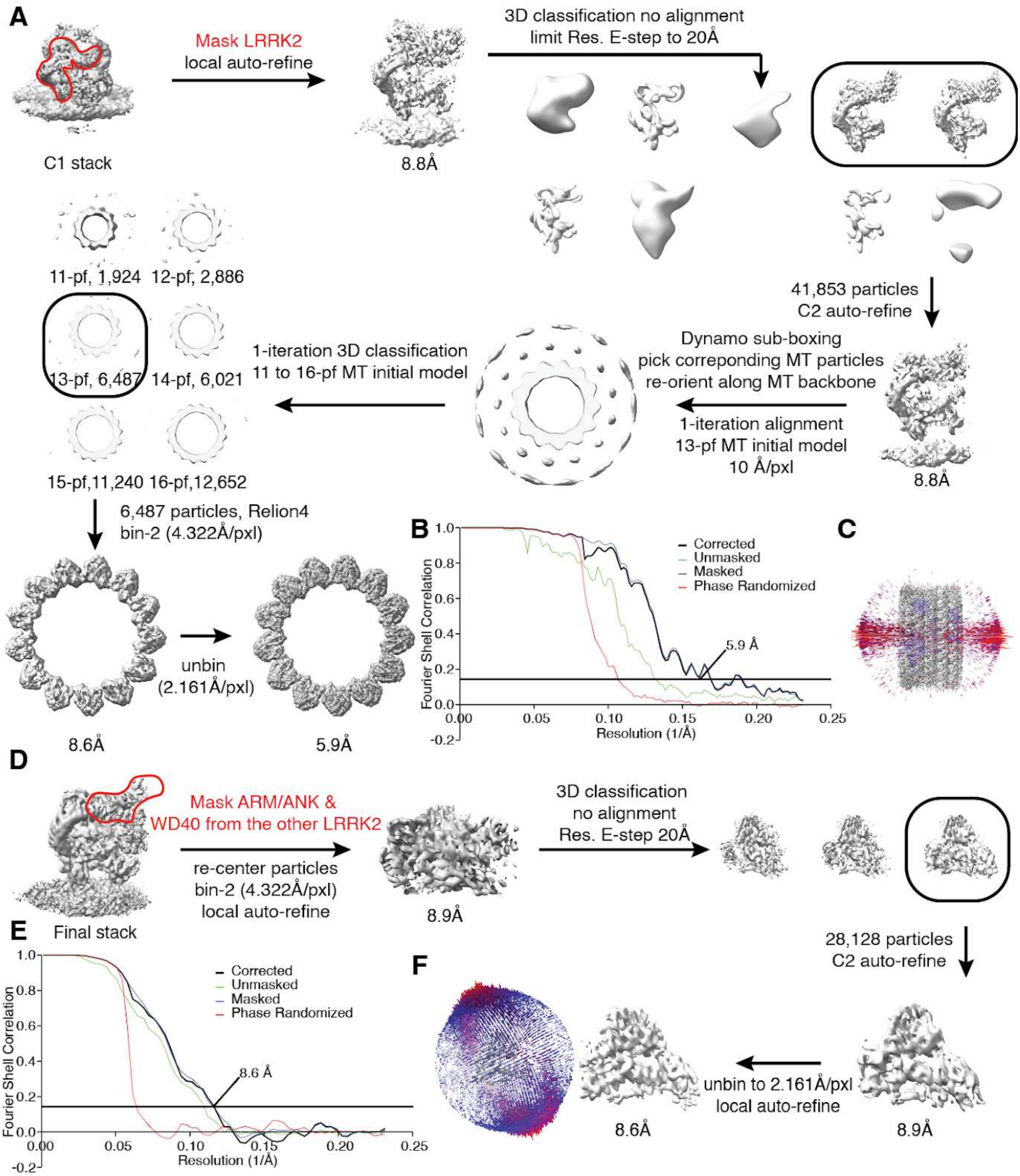

Figure S3: Data-processing scheme for the focused-refined maps from the LRRK2 + MLi-2 + microtubule sample. (A) Flow chart of the cryo-ET data processing procedure from the microtubule-LRRK2 map to obtain the 13-pf microtubule reconstruction map. **(B)** The gold-standard Fourier Shell Correlation (FSC) curves (0.143 cutoff) show the final resolution of the microtubule map. **(C)** Particle projection angle distribution plot of the microtubule map. **(D)** Flow chart of the cryo-ET data processing procedure from the final LRRK2 map to obtain focused map containing the WD40:ARM/ANK interface. **(E)** The gold-standard FSC curves (0.143 cutoff) show the final resolution of the focused refined map. **(F)** Particle projection angle distribution plot of the map.

bioRxiv preprint doi: https://doi.org/10.1101/2024.06.18.599606; this version posted June 20, 2024. The copyright holder for this preprint (which was not certified by peer review) is the author/funder, who has granted bioRxiv a license to display the preprint in perpetuity. It is made available under aCC-BY 4.0 International license.

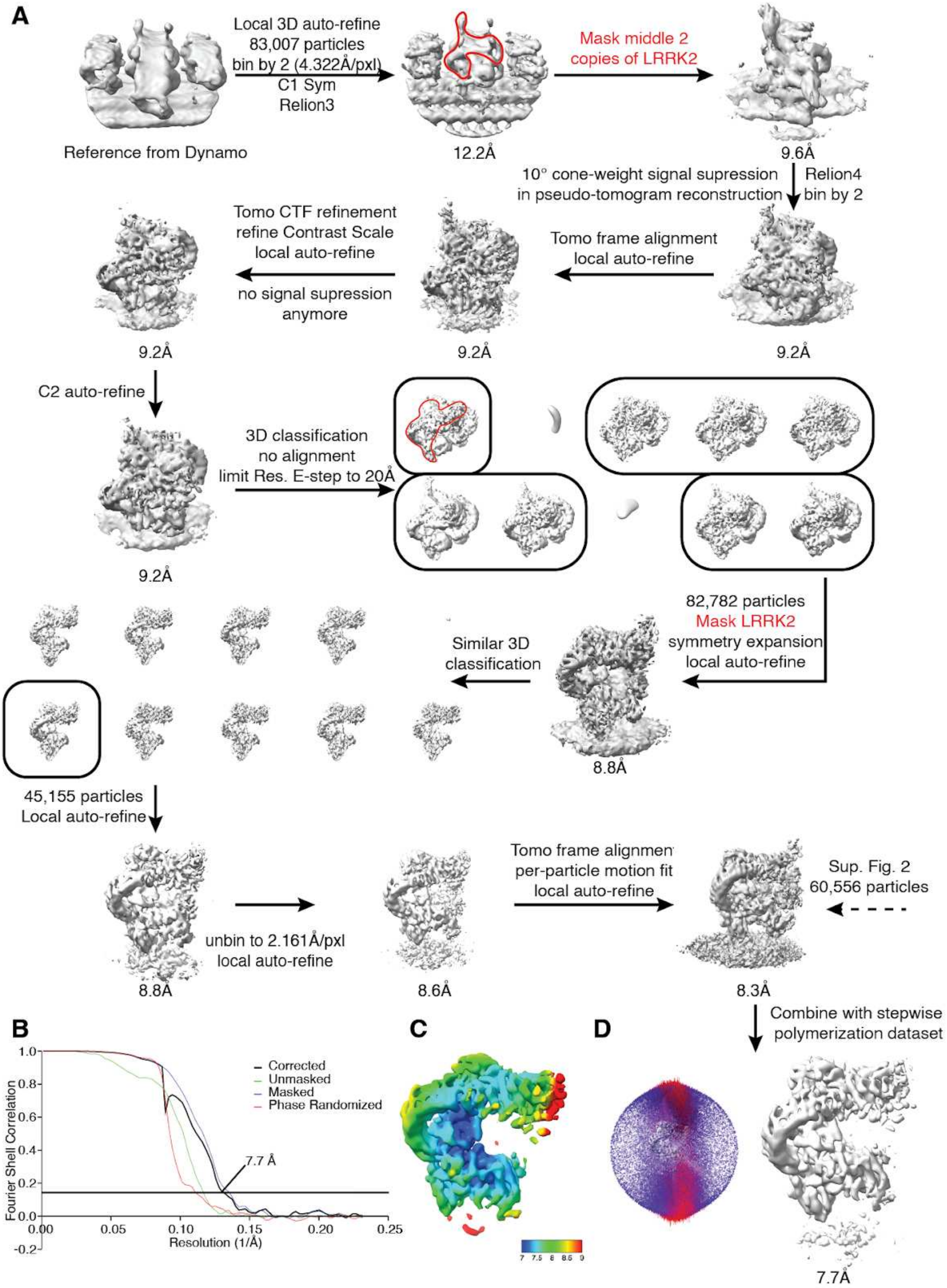

Figure S4: Data-processing scheme for the LRRK2^{I2020T} + MLi-2 + microtubule sample with LRRK2^{I2020T} and microtubule co-polymeriztion. **(A)** Flow chart of the cryo-ET data processing procedure. **(B)** The gold-standard Fourier Shell Correlation (FSC) curves (0.143 cutoff) show the final resolution of the LRRK2 focused-refinement map. **(C)** Local resolution of the LRRK2 focused-refinement map. **(D)** Particle projection angle distribution plot of the map.

bioRxiv preprint doi: https://doi.org/10.1101/2024.06.18.599606; this version posted June 20, 2024. The copyright holder for this preprint (which was not certified by peer review) is the author/funder, who has granted bioRxiv a license to display the preprint in perpetuity. It is made available under aCC-BY 4.0 International license.

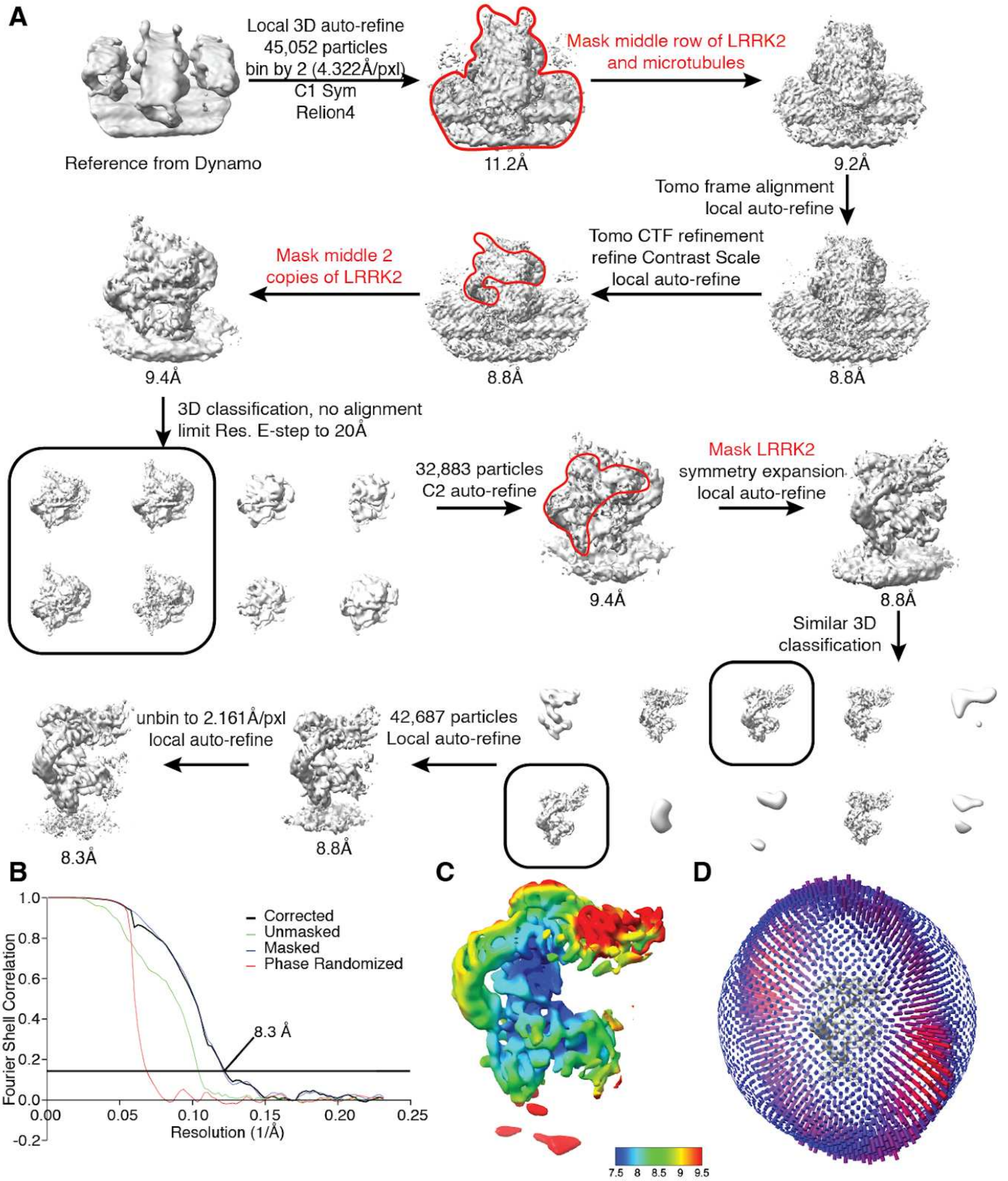

Figure S5: Data-processing scheme for the LRRK2 + MLi-2 + microtubule sample. (A) Flow chart of the cryo-ET data processing procedure. **(B)** The gold-standard Fourier Shell Correlation (FSC) curves (0.143 cutoff) show the final resolution of the LRRK2 focused-refinement map. **(C)** Local resolution of the LRRK2 focused-refinement map. **(D)** Particle projection angle distribution plot of the map.

bioRxiv preprint doi: https://doi.org/10.1101/2024.06.18.599606; this version posted June 20, 2024. The copyright holder for this preprint (which was not certified by peer review) is the author/funder, who has granted bioRxiv a license to display the preprint in perpetuity. It is made available under aCC-BY 4.0 International license.

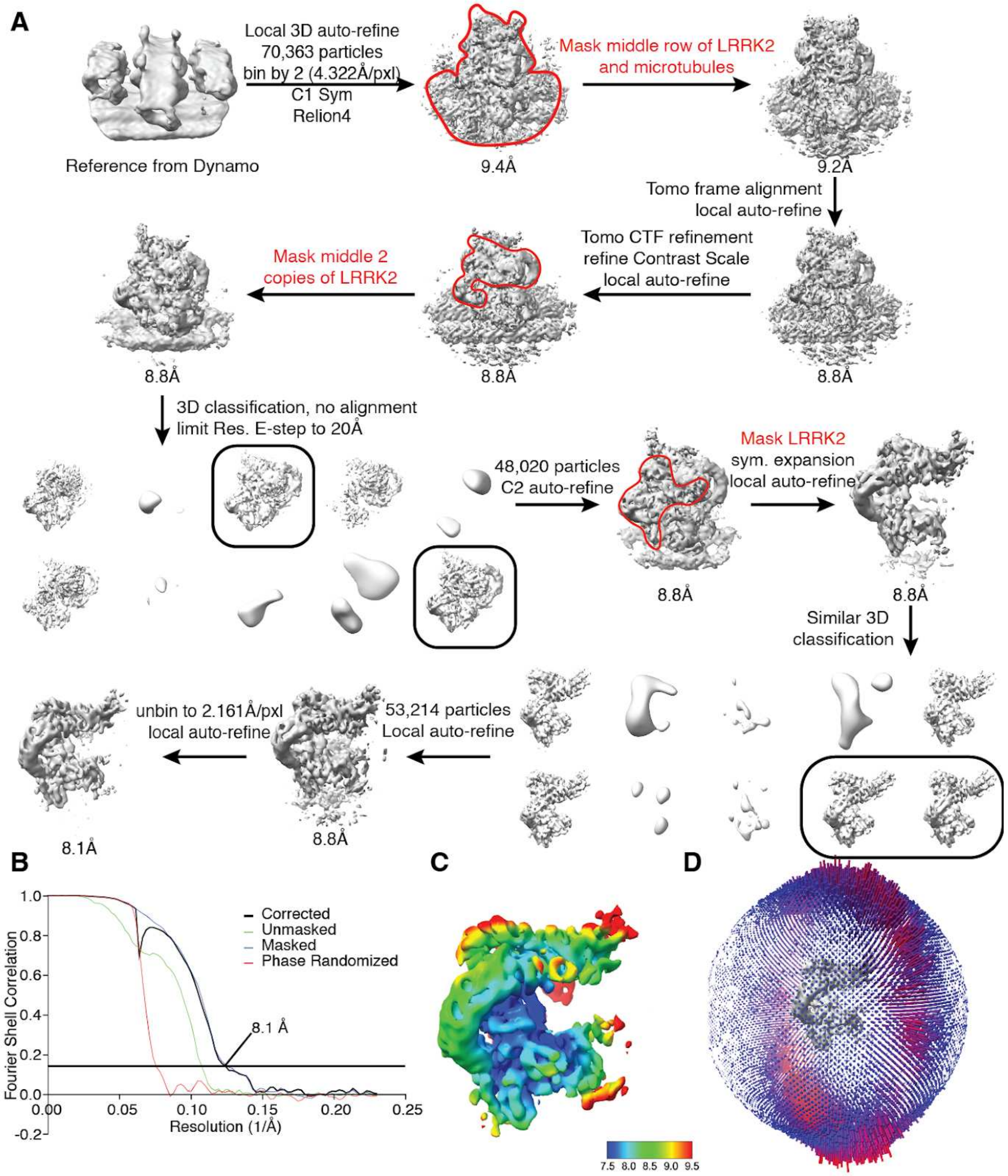

Figure S6: Data-processing scheme for the LRRK2 + GZD-824 + microtubule sample. (A) Flow chart of the cryo-ET data processing procedure. **(B)** The gold-standard Fourier Shell Correlation (FSC) curves (0.143 cutoff) show the final resolution of the LRRK2 focused-refinement map. **(C)** Local resolution of the LRRK2 focused-refinement map. **(D)** Particle projection angle distribution plot of the map.

bioRxiv preprint doi: https://doi.org/10.1101/2024.06.18.599606; this version posted June 20, 2024. The copyright holder for this preprint (which was not certified by peer review) is the author/funder, who has granted bioRxiv a license to display the preprint in perpetuity. It is made available under aCC-BY 4.0 International license.

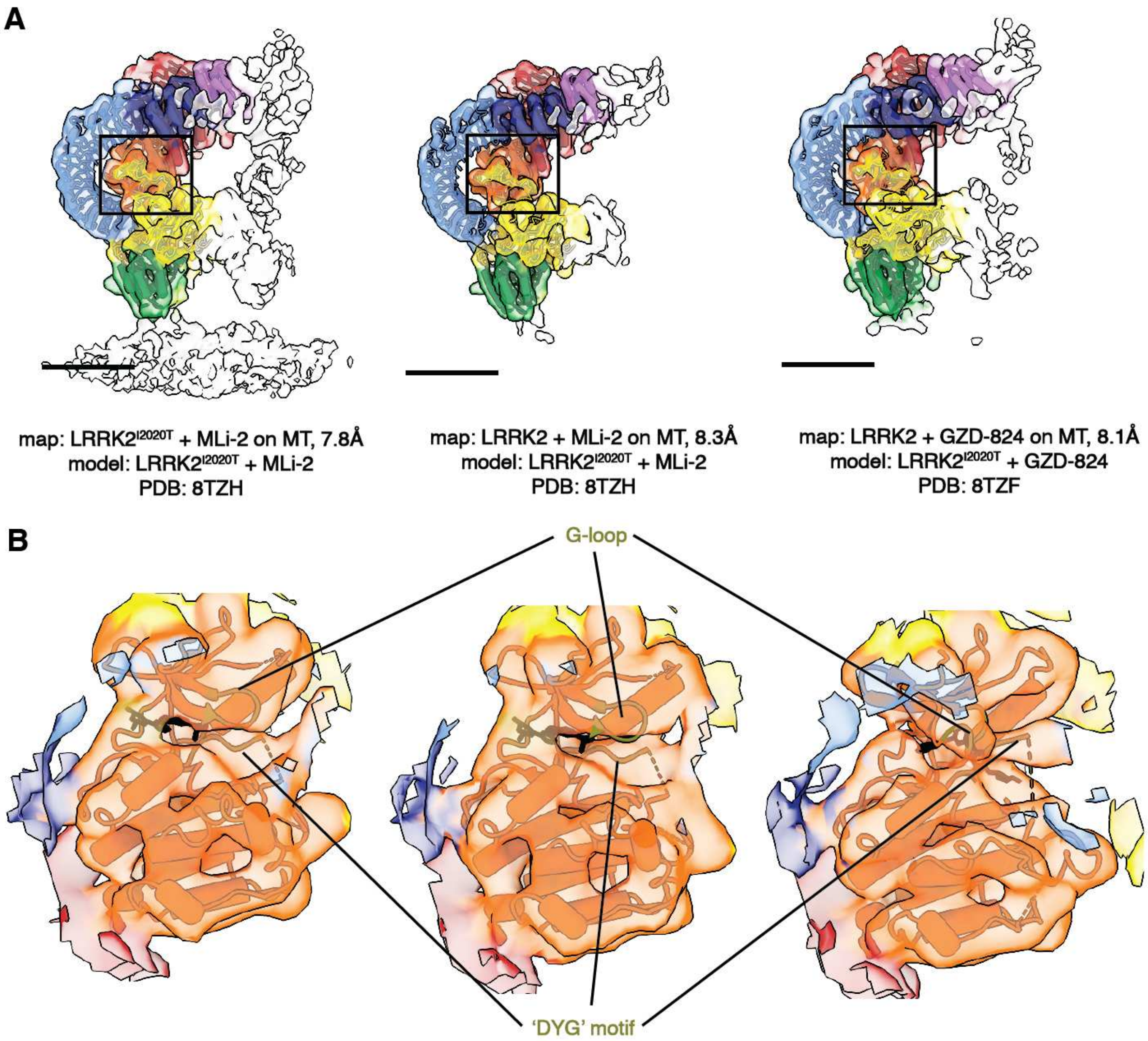

Figure S7: Comparison of the LRRK2 kinase domain conformations in the in-solution and microtubule-bound structures. (A) Models of in-solution LRRK2^{I2020T} bound to kinase inhibitors were fitted into our maps of LRRK2 bound to microtubules. The color scheme is the same one introduced in Fig. 1. The published LRRK2 models that were fitted into each map contain the same kinase inhibitor used in the sample giving rise to the map, as noted below each image. **(B)** Close-up view focusing on the fitting of the kinase from the published LRRK2-inhibitor complexes into each of our LRRK2 maps. The motifs that undergo major conformational changes, the 'G-loop' and the 'DYG' motif, are highlighted and colored in olive green. The inhibitors are shown in black.

bioRxiv preprint doi: https://doi.org/10.1101/2024.06.18.599606; this version posted June 20, 2024. The copyright holder for this preprint (which was not certified by peer review) is the author/funder, who has granted bioRxiv a license to display the preprint in perpetuity. It is made available under aCC-BY 4.0 International license.

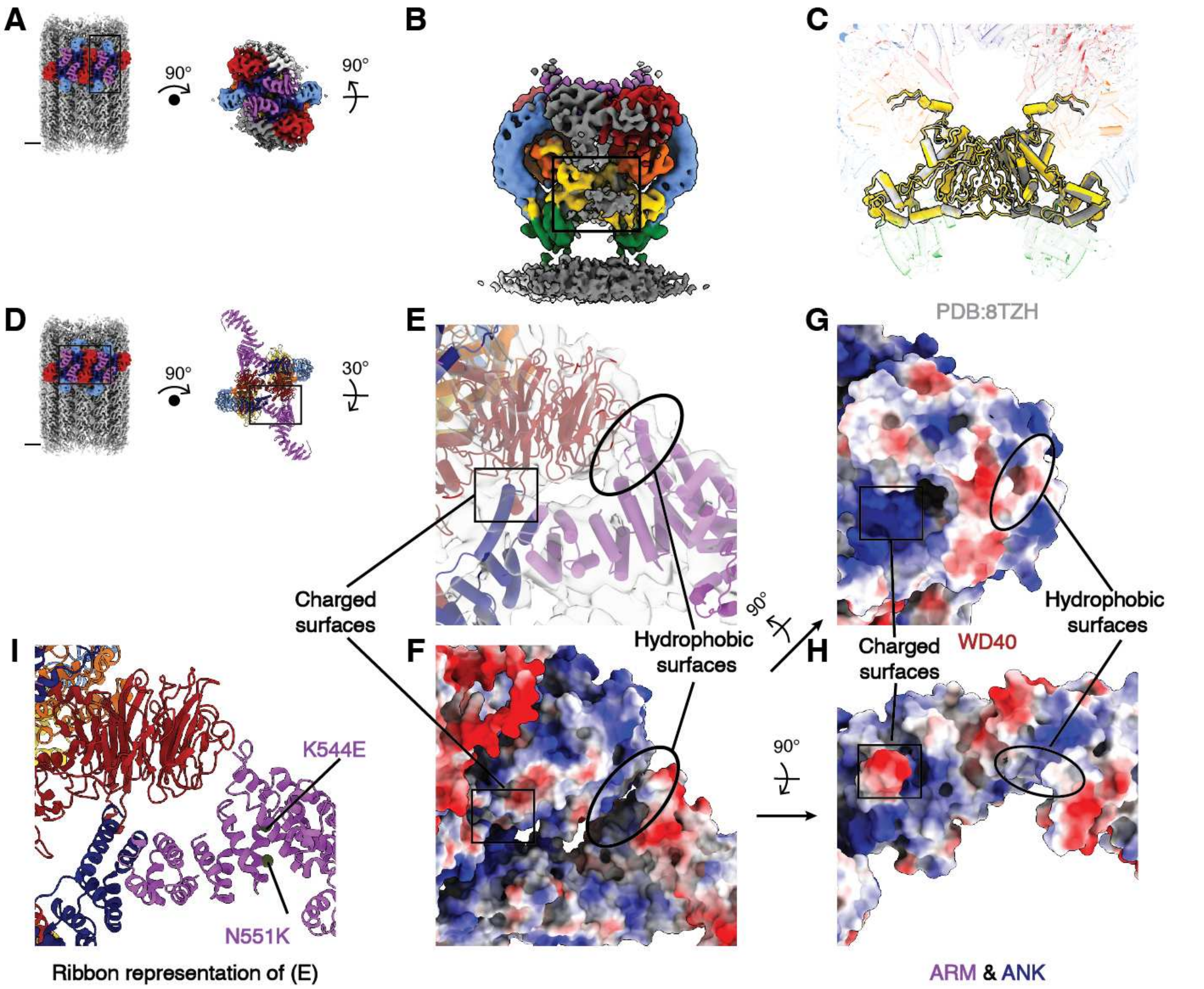

Figure S8: Surface electrostatic potential at the WD40:ARM/ANK interaction interfaces in the LRRK2 WD40 dimer observed in the LRRK2-microtubule complex. (A) The location of the close-up view shown in (B) is indicated by the black square on the composite map (left). How panel (B) is generated by rotating from the composite map is described step-by-step. **(B)** The rotated LRRK2 COR:COR dimer map (colored) derived from the autoinhibited LRRK2 map on microtubules. The viewing angle is the same as in Fig. 1J. **(C)** The LRRK2 in-solution model (light and dark gray) and the autoinhibited LRRK2 model on microtubules (colored) are fitted into the map shown in (B) and superimposed to each other for comparison. The view is focused on the COR domains of both LRRK2 models and the rest of the models are set as transparent in the background. **(D)** The location of the close-up view shown in (E) is indicated by the black square on the composite map (left) and the LRRK2 chimeric WD40:WD40 dimer model (right). How panel (E) is generated by rotating from the composite map is described step-by-step. **(E)** Close-up view of the interaction surface between the ARM/ANK domains and the WD40 domain. The view shown here is identical to Fig. 2G and serves as the reference view. **(F)** The corresponding surface electrostatic potential map aligned to (E). **(G-H)** Surface electrostatic potential view of the ARM/ANK domain (G) and the WD40 domain (H). Both hydrophobic surfaces and charged surfaces are highlighted. **(I)** ribbon representation of (E) showing the PD associated LRRK2 mutations identified from patients.

bioRxiv preprint doi: https://doi.org/10.1101/2024.06.18.599606; this version posted June 20, 2024. The copyright holder for this preprint (which was not certified by peer review) is the author/funder, who has granted bioRxiv a license to display the preprint in perpetuity. It is made available under aCC-BY 4.0 International license.

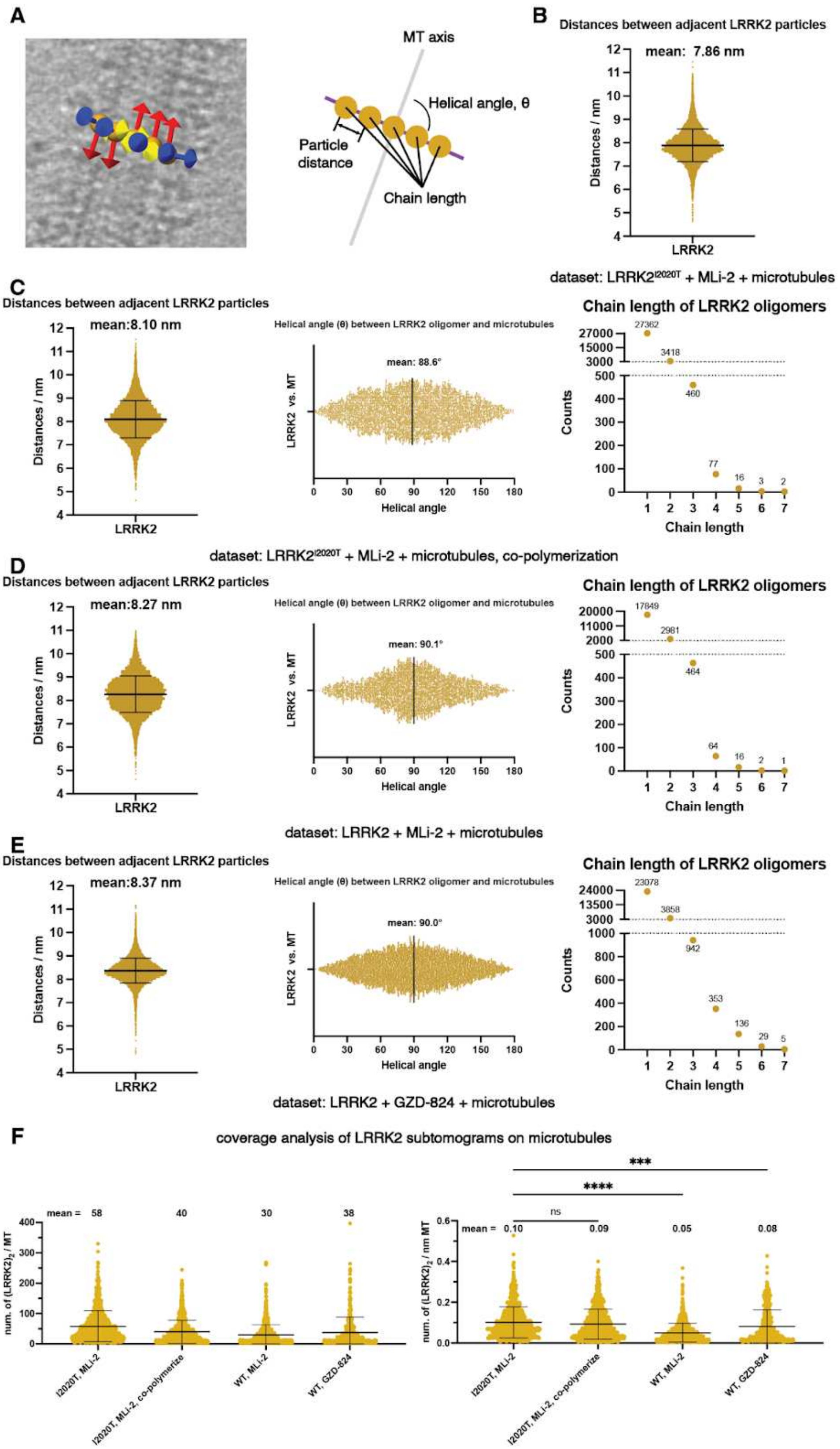

Figure S9: Geometrical analysis of LRRK2 subtomograms for the different LRRK2-microtubule datasets. (A) As in Fig. 3B, subtomogram coordinates (orange spheres) and their refined x-y-z (yellow-red-blue) orientations are mapped back to the original tomogram (left) to illustrate the analyzed geometrical parameters (right). Distances between adjacent particles, helical angles and $(LRRK2)_2$ group chain length is labeled on the cartoon. **(B)** Distribution of distances between adjacent LRRK2 subtomograms. Only subtomogram pairs that fall into the same $(LRRK2)_2$ group are analyzed. The analysis was applied to the same dataset and the same set of $(LRRK2)_2$ groups as in Fig. 3 E-F. LRRK2 subtomograms are grouped based on their relative positions and distances. **(C)** Distribution of distances, helical angles and group chain length for the LRRK2 + MLi-2 + microtubule dataset. **(D-E)** Same as in C, for the LRRK2 + MLi-2 + microtubule dataset (D), and the LRRK2 + GZD-824 + microtubule dataset (E). **(F)** Distribution of $(LRRK2)^2$ subtomogram number along each microtubule (left) and along a fixed length (1 nm) of microtubules (right). The mean ± s.d. and P values (one-way ANOVA, ns:P > 0.05; *:P ≤ 0.05; **:P ≤ 0.01; ***:P ≤ 0.001; ****:P ≤ 0.0001) of the indicated comparisons are shown (n = number of microtubules for each category).

bioRxiv preprint doi: https://doi.org/10.1101/2024.06.18.599606; this version posted June 20, 2024. The copyright holder for this preprint (which was not certified by peer review) is the author/funder, who has granted bioRxiv a license to display the preprint in perpetuity. It is made available under aCC-BY 4.0 International license.

Supplementary Table 1: Cryo-ET data collection and model statistics

	#1 LRRK2^{I2020T}+MLi-2, step-wise polymerization (EMDB-xxxxx: LRRK2) (EMDB-xxxxx: microtubule) (EMDB-xxxxx: combined with dataset #2) (PDB xxxx)	#2 LRRK2^{I2020T}+MLi-2, co-polymerization (EMDB-xxxxx: LRRK2) (EMDB-xxxxx: LRRK2 WD40 dimer with microtubule)	#3 LRRK2 + MLi-2 (EMDB-xxxxx: LRRK2)	#4 LRRK2 + GZD-824 (EMDB-xxxxx: LRRK2)
**Data collection and processing**				
Magnification	42k			
Voltage (kV)	300			
Total dose (e–/Å^2)	120			
Defocus range (µm)	-3.0 to -5.0			
Defocus increment	0.5			
Acquisition scheme	Dose-Symmetric, -54/54, 3° step, group 3			
Pixel size (Å)	2.161			
No. of frames	8			
# of tomograms	131	150	196	143
# of subtomograms	65,612	83,007	45,052	70,363
Final particle #	60,556	45,155	42,687	53,214
Symmetry imposed	C2 for LRRK2 WD40 dimer, C1 for symmetry expanded LRRK2, -27.7° rot and 9.4 Å rise for microtubule			
Map resolution (Å) FSC threshold = 0.143 (Å)	7.8 (LRRK2), 5.9 (microtubule), 7.7 (LRRK2, combined)	8.3 (LRRK2), 8.8 (LRRK2 dimer and microtubule)	8.3 (LRRK2)	8.1 (LRRK2)
Map resolution range (Å)	7 to >9	7.5 to >9.5	7.5 to >9.5	7 to >9
**Refinement**				
Initial model used (PDB code)	7LHT, 8TZH			
Model resolution (Å) FSC threshold = 0.143 (Å)	7.8 (LRRK2)			
Model resolution range (Å)	n/a			
Map sharpening *B* factor (Å^2)	-653 (LRRK2)			
Model composition				
Non-hydrogen atoms	26,693			
Protein residues	1,792			
Ligands	MLi-2:1, GDP:1			
B factors (Å^2)				
Protein	471.87			
Ligand	N/A			
R.m.s. deviations				
Bond lengths (Å)	0.021			
Bond angles (°)	1.419			
Validation				
MolProbity score	2.20			
Clashscore	14.23			
Poor rotamers (%)	0.06			
Ramachandran plot				
Favored (%)	90.17			
	9.83			

bioRxiv preprint doi: https://doi.org/10.1101/2024.06.18.599606; this version posted June 20, 2024. The copyright holder for this preprint (which was not certified by peer review) is the author/funder, who has granted bioRxiv a license to display the preprint in perpetuity. It is made available under aCC-BY 4.0 International license.

Allowed (%)	0.00
Disallowed (%)	
